# Decomposing the rural-urban disparities in overweight and obesity among women of reproductive age in Nigeria

**DOI:** 10.1186/s12905-023-02813-2

**Published:** 2023-12-21

**Authors:** Ololade Julius Baruwa, Babatunde Makinde Gbadebo, Oluwafemi John Adeleye, Hanani Tabana, Adeniyi Francis Fagbamigbe

**Affiliations:** 1 School of Public Health, University of the Western Cape, Cape Town, South Africa; 2 Department of Epidemiology and Medical Statistics, Faculty of Public Health, College of Medicine. University of Ibadan, Ibadan, Nigeria

**Keywords:** Obesity, Overweight, Sociodemographic, Nigeria, Women

## Abstract

**Background:**

Overweight and obese women face various reproductive and other health challenges, and in some cases, even mortality. Despite evidence of rural-urban disparities in overweight and obesity among women of reproductive age, there is limited evidence regarding the predictors of these disparities. This study aims to investigate the factors associated with overweight and obesity and examine the contributors to rural-urban disparities in overweight and obesity among women of reproductive age in Nigeria.

**Methods:**

We utilized the 2018 Nigeria Demographic and Health Survey dataset. The survey employed a two-stage cluster sampling technique based on Nigeria’s 2006 census enumeration areas for sample selection. Overweight and obesity were defined as a body mass index (BMI) ≥ 25. Data analyses were conducted using the Logistic Regression Model and the threefold Blinder-Oaxaca decomposition model (α0.05).

**Results:**

The study revealed that older women (OR = 2.44; CI = 2.11–2.83), those with higher wealth (OR = 2.05; CI = 1.81–2.31), contraceptive users (OR = 1.41; CI = 1.27–1.57), and residents of the South-South region (OR = 1.24; CI = 1.07–1.45) were more likely to be overweight/obese. The decomposition analysis indicated that the mean predicted prevalence of overweight and obesity is 35.5% in urban areas, compared to 21.1% in rural areas of Nigeria. Factors such as wealth status, educational level, media exposure, and contraceptive use were identified as significant contributors to these disparities.

**Conclusion:**

The findings underscore the importance of addressing socioeconomic disparities when designing healthcare interventions to reduce the burden of overweight and obesity, particularly in urban areas. Prioritizing these factors can facilitate efforts to promote healthier lifestyles and enhance overall well-being.

## Introduction

Overweight and obesity pose significant global public health threats, particularly to women’s health. Over the past three decades, their prevalence has surged across all age groups [1]. In 2016, over 1.9 billion adults were reported as overweight, with 650 million classified as obese [1]. Projections estimate that by 2030, the number of people with obesity will reach 1.12 million [2]. What was once considered primarily a concern in Western nations has now escalated in developing countries, with Africa experiencing substantial increases [3]. Sub-Saharan African (SSA) women bear a significant burden, with a 2010 World Health Organization (WHO) estimate, indicating a high prevalence of overweight at 73.8% and obesity at 43.2% [4]. Nigeria is no exception to this trend, with a notable prevalence of overweight and obesity among its women [4, 5]. In fact, women exhibit a slightly higher prevalence of overweight compared to men, with 25.5% versus 25.2 and 19.8% versus 12.9% for obesity, respectively [6].

The variation and disparity of overweight and obesity between rural and urban communities is a prevalent global public health concern and holds true for several countries, including Nigeria. For example, in Zambia, the prevalence of overweight and obesity was 21.2 and 10.8% among urban women versus 11.9 and 2.9% among rural women (7, Bwalya, 2017). In Nigeria, similar disparities are observed, with rural and urban areas experiencing contrasting prevalence rates. The prevalence of obesity in urban areas is significantly higher, reaching 16.5%, whereas rural areas report a lower prevalence of 4.0% [8]. Overweight and obesity are complex conditions influenced by multiple factors, including dietary habits [9–11], physical activity and lifestyle [12, 13], genetics [14], psychological factors [15, 16], environmental factors [17, 18], and medical conditions such as hormonal imbalances [9].

Women with overweight and obese face diverse reproductive and other health challenges and even deaths in some cases [6, 19]. Reproductive disorders linked to overweight, and obesity include menstrual irregularities, anovulation, infertility, and adverse pregnancy outcomes such as miscarriages [20]. Moreover, being overweight and obese increases the likelihood of developing type 2 diabetes, hypertension, coronary heart disease, obstructive sleep apnea, osteoarthritis, and various cancers (e.g., ovarian, breast, prostate, endometrial, liver, kidney, gallbladder, and colon cancers) [6, 21, 22]. Being overweight and obese also contributes to a higher incidence of non-communicable diseases and is associated with orthopedic issues and back pain [3, 23, 24]. These conditions adversely affect contraception and fertility, with maternal overweight and obesity being linked to an increased risk of cesarean sections and obstetric complications like diabetes and hypertension [25]. Furthermore, maternal overweight and obesity have detrimental effects on pregnancy outcomes, including an increased risk of neonatal death and malformations, reduced breastfeeding initiation and duration, and various health challenges for children born to overweight or obese mothers [26, 27].In addition, the economic burden of overweight and obesity is immense [28, 29], putting substantial pressure on healthcare facilities [30].

Numerous efforts have been initiated to combat overweight and obesity due to their detrimental impact on women’s health [31]. Nigeria has joined this global effort. However, despite improvements in healthcare and interventions, the decline in overweight and obesity rates among reproductive-aged women is progressing slowly, hindering the achievement of Sustainable Development Goal 3 (SDG 3). Addressing this challenge requires comprehensive strategies and collaborative efforts from both the government and society to significantly curb the growth of overweight and obesity in the country. Investigating disparities in overweight and obesity, particularly among women of reproductive age, is crucial for guiding effective intervention strategies. Several epidemiological studies have explored overweight and obesity among women of reproductive age in Nigeria [4, 5, 32], but gaps in their outcomes exist. To our knowledge, no study has examined the predictors of rural-urban disparities in overweight and obesity among women of reproductive age in Nigeria using the nationally representative data from the 2018 Nigeria Demographic and Health Survey (NDHS). This study seeks to contribute to ongoing discussions aimed at addressing the challenges associated with overweight and obesity among women of reproductive age in Nigeria and enhancing their overall well-being. Specifically, the study investigates factors associated with overweight and obesity and explores the contributors to rural-urban disparities in overweight and obesity among women of reproductive age in Nigeria. The findings from this study will provide insights into factors that need to be considered when designing strategies and policies to mitigate overweight and obesity in Nigeria.

## Methods

The data for this study were extracted from the 2018 Nigeria Demographic and Health Survey (NDHS) individual (women) dataset. The 2018 NDHS was conducted by the National Population Commission of Nigeria and ICF (International). The survey specifically collected nationally representative data on background characteristics, birth history, antenatal care, contraceptive use, women’s dietary diversity, and domestic violence, among other factors. The survey employed a two-stage cluster sampling technique based on Nigeria’s 2006 census enumeration areas for sample selection. A total of 42,121 women were eligible to participate in the women’s individual interviews; however, 41,821 women were successfully interviewed, resulting in a response rate of 99%. The study design was cross-sectional, and the unit of analysis included women between the ages of 15–49 years. Detailed information on sample size estimation and sampling strategies used for data collection is available in the full NDHS published reports, accessible to the public at (https://www.dhsprogram.com/). Women who were pregnant at the time of the survey and those with missing Body Mass Index data were excluded from the analysis. After data wrangling and cleaning, the unit of analysis for this study was reduced to 13,339 individuals.

## Outcome variable

The outcome variable in the current study is overweight and obesity, which is assessed using Body Mass Index (BMI). Prior studies have consistently used this measurement [33, 34]. Body Mass Index is defined as an individual’s weight in kilograms divided by the square of their height in meters. According to the World Health Organization’s (WHO) international standard definition, a BMI less than 18.5 is categorized as underweight, a BMI between 18.5 and 24.99 falls within the range of normal weight, a BMI between 25 and 29.99 indicates overweight, and a BMI equal to or greater than 30 kg/m^2^ is classified as obesity [1]. In this study, our outcome variable is having a BMI equal to or greater than 25, which is coded as “1,” and “0” is used for all other cases.

## Independent variable

The selection of explanatory variables in this study was guided by prior research in the literature. Specifically, we considered independent variables for which there is empirical evidence supporting their relationship with overweight and obesity [7, 12, 35, 36]. One of the independent variables of primary interest in this study includes household wealth status, which is approximated using the wealth quintile as a proxy. Wealth status is a crucial social determinant of health and is often associated with various health outcomes, including overweight and obesity [37]. It plays a significant role in examining socioeconomic disparities and intersects with other social determinants of health, such as gender, race, educational level, and environmental factors, influencing health outcomes in complex ways [17, 38].

Additionally, other explanatory variables included in this study are age, categorized into three groups (15–24, 25–34, and 35–49), education (measured as no education, primary, secondary, and tertiary), marital status (measured as never married, currently married, and previously married), media exposure (assessed by ever reading a newspaper, watching television, or listening to the radio, categorized as exposed and unexposed), religion, employment status, contraceptive use, the number of living children, place of residence, and region. These variables were selected based on their relevance in the existing literature and their potential influence on overweight and obesity.

## Data analysis

The data for this study were weighted to guarantee the representativeness of the survey. We utilized Stata version 16 software for both descriptive and inferential analyses. The Chi-square test and multivariable logistic regression analysis were conducted on the data. The Chi-square test served the purpose of establishing relationships between the set of independent variables and the outcome. Subsequently, we conducted further analysis to identify the factors associated with overweight and obesity using a logistic regression model. We also examined rural-urban differences in overweight and obesity while considering other independent variables. The decomposition analysis was performed using the Oaxaca-Blinder decomposition model. All statistical analyses were considered significant at a 5% level of significance.

### Logistic regression model

Logistic regression, a widely employed statistical model, is particularly suited for modeling binary categorical variables. It excels in determining the relationship between a set of predictor variables and a categorical response variable by estimating probabilities [39]. In logistic regression, each independent variable is assigned a coefficient that measures its individual contribution to the variation in the outcome variable. This model, based on individual characteristics, uses natural logarithms and odds ratios to predict the probabilities of being overweight and obese.

1 $$\ln \left[\frac{P(Y)}{1-P(Y)}\right]={\beta}_0+{\beta}_1{X}_1+{\beta}_2{X}_2+\dots +{\beta}_n{X}_n$$

2 $$\frac{P(Y)}{1-P(Y)}=\exp \left({\beta}_0+{\beta}_1{X}_1+{\beta}_2{X}_2+\dots +{\beta}_n{X}_n\right)$$

3 $$P(Y)=\frac{\exp \left({\beta}_0+{\beta}_1{X}_1+{\beta}_2{X}_2+\dots +{\beta}_n{X}_n\right)}{1+\exp \left({\beta}_0+{\beta}_1{X}_1+{\beta}_2{X}_2+\dots +{\beta}_n{X}_n\right)}$$

Where, $\ln \left[\frac{P(Y)}{1-P(Y)}\right]$ is the log (odds) of overweight and obesity, P(Y) is the probability of the binary outcome (overweight and obesity), *X*_1_, *X*_2_,…, *X*_*n*_ are the predictor variables, *β*_1_, *β*_2_, …, *β*_*n*_ are the coefficient of the predictors and *β*_0_ is the intercept. The regression coefficients indicate the magnitude of the relationship between each predictor variable and the outcome [40]. All predictor variables empirically relevant to predicting overweight and obesity have been included in the model. The analysis results are presented in terms of odds ratios, along with their corresponding 95% confidence intervals.

### Blinder Oaxaca decomposition model

The Blinder-Oaxaca decomposition model is a widely utilized technique for identifying and quantifying distinct contributions of group differences in measurable characteristics, such as education, marital status, wealth status, and regional disparities, to variations in wealth, racial, and gender gaps in outcomes. It extends the original decomposition technique introduced by Fairlie [41]. This technique derives its coefficient estimates from the logit regression model, following the calculation of the difference (inequality) in the mean outcome between two groups. The model elucidates the extent to which the observed difference is attributable to disparities in the levels of observed characteristics and how much is attributed to discrimination. This discrimination may arise from differential effects of observed characteristics and other unknown associated factors [42–44]. The model dissects the predicted rural-urban differences in overweight and obesity, generating three components: the difference due to the endowment effect, the difference due to the coefficient effect, and interaction.

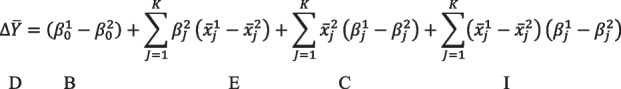



Where, $\Delta \overline{Y}$ is the predicted rural-urban difference (D) in overweight and obesity; $\left({\beta}_0^1-{\beta}_0^2\right)$ is the effect of unobservable variables not taken into account (B); $\sum_{J=1}^K{\beta}_J^2\left({\overline{x}}_j^1-{\overline{x}}_j^2\right)$ is the portion of the difference (D) that is explained by group difference in the level of observable explanatory variables also known as endowments effect (E); $\sum_{J=1}^K{\overline{x}}_j^2\left({\beta}_j^1-{\beta}_j^2\right)$ arises from the differential effect of the explanatory variables also known as the coefficient effect (C) and $\sum_{J=1}^K\left({\overline{x}}_j^1-{\overline{x}}_j^2\right)\left({\beta}_j^1-{\beta}_j^2\right)$ is the interaction (I) due to simultaneous effect of the differences in endowments and coefficients.

## Result

The distribution of respondents by background characteristics is presented in Table 1. The results reveal that 35.3% of the respondents are categorized as poor, while the majority (35%) of women fall within the age group of 15–24 years. Table 1 also indicates that 31.0% of the respondents have no formal education, 68.2% are currently married, 52.5% identify as Christians, and 66.1% are employed. Furthermore, the table shows that 31.4% of the respondents have not been exposed to media, 83.1% use contraceptives, 42.1% reside in urban areas, and 18.6% live in the North Central region.

**Table 1:** Percentage distribution of the respondents according to background characteristics

Background Characteristics	(*N* = 13,339) Frequency (n)	Percentage (%)
**Household poverty-wealth**		
Poor	4716	35.3
Middle	2954	22.2
Rich	5669	42.5
**Age**		
15–24	4665	35.0
25–34	4216	31.6
35–49	4458	33.4
**Education**		
None	4136	31.0
Primary	2153	16.1
Secondary	5669	42.5
Tertiary	1381	10.4
**Marital Status**		
Never Married	3434	25.7
Currently Married	9093	68.2
Previously Married	812	6.1
**Religion**		
Christianity	7005	52.5
Muslim	6210	46.6
Other	124	0.9
**Employment Status**		
Unemployed	4527	33.9
Employed	8812	66.1
Media Exposure		
Not Exposed	4185	31.4
Exposed	9154	68.6
**Contraceptive Use**		
User	11,080	83.1
None-User	2259	16.9
**Number of Living Children**		
None	3633	27.2
One	1663	12.5
Two	1674	12.6
Three	1577	11.8
Four	1504	11.3
Five and above	3288	24.7
**Place of Residence**		
Urban	5611	42.1
Rural	7728	57.9
**Region**		
North Central	2487	18.6
North East	2260	16.9
North West	2809	21.1
South East	1930	14.5
South South	1788	13.4
South West	2065	15.5

## Prevalence of overweight and obesity by background characteristics of the respondents

Table 2 presents the prevalence of overweight and obesity by background characteristics in 2018. The table reveals that the prevalence of overweight and obesity among women of reproductive age is 27.2%. Approximately, 11.5% of respondents aged 15–24 are classified as overweight or obese, which is significantly lower than the percentages for the age group 35–49 (41.2%). In terms of education, the prevalence of overweight and obesity is highest among respondents with tertiary education (48.2%), followed by primary education (30.1%).

**Table 2:** Percentage distribution of the respondents according to overweight and obesity by background characteristics, 2018

Background Characteristics	Overweight and Obesity			
	Frequency (n)	Percentage (%)	*χ*^2^	*p*-value
**Total**	3628	27.2		
**Household poverty-wealth**				
Poor	617	13.1	988	< 0.001
Middle	719	24.3		
Rich	2292	40.4		
**Age**				
15–24	535	11.5	1039.1	< 0.001
25–34	1256	29.8		
35–49	1837	41.2		
**Education**				
None	653	15.8	600.2	< 0.001
Primary	648	30.1		
Secondary	1662	29.3		
Tertiary	665	48.2		
**Marital Status**				
Never Married	528	15.4	346.9	< 0.001
Currently Married	2791	30.7		
Previously Married	309	38.1		
**Religion**				
Christianity	2467	35.2	488.8	< 0.001
Muslim	1123	18.1		
Other	38	30.7		
**Occupation Status**				
Unemployed	826	18.3	277.4	< 0.001
Employed	2802	31.8		
**Media Exposure**				
Not Exposed	630	15.1	454.3	< 0.001
Exposed	2998	32.8		
**Contraceptive use**				
User	2638	23.8	379.7	< 0.001
None-User	990	43.8		
**Number of living Children**				
None	561	15.4	397.5	< 0.001
One	426	25.6		
Two	504	30.1		
Three	509	32.3		
Four	536	35.6		
Five and above	1092	33.2		
**Place of Residence**				
Urban	1994	35.5	340.1	< 0.001
Rural	1634	21.1		
**Region**				
North Central	674	27.1	646.2	< 0.001
North East	358	15.8		
North West	437	15.6		
South East	716	37.1		
South South	705	39.4		
South West	738	35.7		

Concerning marital status, the prevalence of overweight and obesity is highest among previously married respondents (38.1%), and lowest among never-married respondents (15.4%). Christianity has the highest percentage of overweight and obese respondents (35.2%), followed by other religions (30.7%), and Muslims (18.1%). Employment status also shows a significant association with overweight/obesity, with a higher prevalence among employed respondents (31.8%) compared to unemployed respondents. Similarly, wealth status is significantly associated with overweight/obesity, with a higher prevalence among the rich (40.4%) compared to the poor (13.1%). Media exposure is significantly associated with overweight/obesity, as the prevalence among exposed respondents is higher (32.8%) compared to those not exposed (15.1%). The use of contraceptives likewise demonstrates a significant association, with a higher prevalence of overweight and obesity among non-users (43.8%) compared to users (23.8%).

The prevalence of overweight and obesity increases with the number of living children. For respondents with no children, the prevalence is 15.4%, while it increases to 35.6% for those with four children. Place of residence is also significantly associated with overweight/obesity, with a higher prevalence among urban residents (35.5%) compared to rural residents (21.1%). Lastly, there are regional differences, with the highest prevalence observed in the South-South (39.4%), South East (37.1%), and South West (35.7%), while the lowest prevalence is in the North East (15.8%) and North West (15.6%) regions. The chi-square test shows a significant association between the socio-demographic characteristics of the respondents and overweight/obesity (*p* < 0.001).

### Multivariate logistic regression of determinants of overweight and obesity

The unadjusted and adjusted logistic regression model of overweight and obesity among women of reproductive age is presented in Table 3. Overweight and obesity were significantly predicted by age, education, marital status, religion, occupation status, wealth status, media exposure, contraceptive use, number of living children, place of residence, and region. Age was identified as a significant predictor of overweight and obesity. When compared to women in the 15–24 age category, the odds of being overweight and obese, while controlling for other characteristics, were aOR = 2.38 (95% CI: 2.05–2.77, *p* < 0.001) times higher among women aged 25–34 years and 4.42 (3.74–5.23, *p* < 0.001) times higher among women aged 35–49 years.

**Table 3:** Unadjusted and adjusted logistic regression model of determinants of overweight and obesity among women of reproductive age in Nigeria, 2018

Background Characteristics	Unadjusted Odds Ratio uOR (95%C.I)	Adjusted Odds Ratio aOR (95%C.I)
**Household poverty-wealth**		
Poor	1	1
Middle	2.14 (1.90–2.41)***	1.62 (1.42–1.85)***
Rich	4.51 (4.08–4.98)***	2.74 (2.38–3.14)***
**Age**		
15–24	1	1
25–34	3.28 (2.93–3.66)***	2.38 (2.05–2.77)***
35–49	5.41 (4.86–6.03)***	4.42 (3.74–5.23)***
**Education**		
None	1	1
Primary	2.30 (2.03–2.60)***	1.20 (1.04–1.39)**
Secondary	2.21 (2.00–2.45)***	1.31 (1.12–1.52)***
Tertiary	4.95 (4.33–5.67)***	1.86 (1.55–2.24)***
**Marital Status**		
Never Married	1	1
Currently Married	2.44 (2.20–2.70)***	1.52 (1.24–1.86)***
Previously Married	3.38 (2.85–4.01)***	1.66 (1.29–2.13)***
**Religion**		
Christianity	1	1
Muslim	0.41 (0.37–0.44)***	0.72 (0.63–0.81)***
Other	0.81 (0.55–1.19)	0.97 (0.64–1.48)
**Employment Status**		
Unemployed	1	1
Employed	2.09 (1.91–2.28)***	1.06 (0.96–1.18)
**Media Exposure**		
Not Exposed	1	1
Exposed	2.75 (2.5–3.02)***	1.28 (1.24–1.54)***
**Contraceptive use**		
None-User	1	1
User	2.5 (2.27–2.74)***	1.39 (1.24–1.54)***
**Number of Living Children**		
None	1	1
One	1.89 (1.64–2.17)***	1.10 (0.90–1.36)
Two	2.36 (2.06–2.71)***	1.00 (0.80–1.25)
Three	2.61 (2.27–3.00)***	0.93 (0.74–1.17)
Four	3.03 (2.64–3.48)***	1.05 (0.83–1.32)
Five and above	2.72 (2.43–3.06)***	1.05 (0.84–1.32)
**Place of Residence**		
Urban	1	1
Rural	0.49 (0.45–0.53)***	0.80 (0.73–0.88)***
**Region**		
North Central	1	1
North East	0.51 (0.44–0.58)***	0.82 (0.69–0.96)***
North West	0.50 (0.43–0.57)***	0.81 (0.69–0.95)***
South East	1.59 (1.40–1.80)***	0.90 (0.77–1.06)
South South	1.75 (1.54–1.99)***	1.19 (1.02–1.38)***
South West	1.50 (1.32–1.70)***	0.78 (0.68–0.90)***

***Significant at 1%, **Significant at 5%, *Significant at 10%; *OR* Odds ratio; *CI* Confidence interval

Education level also demonstrated a significant association with overweight and obesity. When compared to women with no formal education, the odds of being overweight and obese were 1.31 (1.13–1.52, *p* < 0.001) times higher among women with secondary education and 1.86 (1.55–2.24, *p* < 0.001) times higher among women with tertiary education. Marital status was found to be a significant predictor as well. Compared to never-married women, the odds of being overweight and obese were 1.52 (1.24–1.86, *p* < 0.001) times higher among currently married women and 1.66 (1.29–2.13, *p* < 0.001) times higher among previously married women.

Religion displayed a significant association with overweight and obesity. When compared to women practicing Christianity, the odds of being overweight and obese were 0.72 (0.63–0.81, *p* < 0.001) times lower among women practicing Islam, while no significant association was observed among women practicing other religions. Occupation status did not show a significant association with overweight and obesity after adjusting for other factors.

Wealth status was found to be a significant predictor of overweight and obesity. When compared to women in the poor wealth status category, the odds of being overweight and obese were 1.62 (1.42–1.85, *p* < 0.001)) times higher among women in the middle wealth status category and 2.74 (2.38–3.14, *p* < 0.001) times higher among women in the rich wealth status category. Media exposure demonstrated a significant association between overweight and obesity. Compared to women not exposed to media, the odds of being overweight and obese were 1.28 (1.14–1.43, *p* < 0.001) times higher among women exposed to media.

Contraceptive use was significantly associated with overweight and obesity. When compared to non-users of contraceptives, the odds of being overweight and obese were 1.39 (1.22–1.54, *p* < 0.001) times higher among contraceptive users. The number of living children did not show a significant association between overweight and obesity after adjusting for other factors. Place of residence was found to be a significant determinant of overweight and obesity. When compared to women living in urban areas, the odds of being overweight and obese were 0.80 (0.73–0.88, *p* < 0.001) times lower among women living in rural areas.

The region also displayed a significant association with overweight and obesity. When compared to women in the North Central region, the odds of being overweight and obese were 0.82 (0.69–0.96, *p* < 0.001) times lower in the North East and 0.78 (0.68–0.90, *p* < 0.001) times lower in the South West region, but 1.19 (1.02–1.38, *p* < 0.001) times higher in the South-South region.

## Decomposition analysis result of rural-urban disparity in overweight and obesity

Table 4 provides an overview of the decomposition analysis results, while Table 5 offers a detailed breakdown of the factors contributing to these disparities. Table 4 illustrate the prevalence of overweight and obesity among urban and rural women. The mean predicted prevalence was 35.5% for urban women and 21.1% for rural women, resulting in a disparity of 14.4%. The decomposition analysis separates this disparity into three components: endowment (E), coefficient (C), and interaction effects.

**Table 4:** Overall decomposition analysis result of rural-urban disparity in overweight and obesity among women of reproductive age in Nigeria - 2018

Overweight and Obesity	Coefficient (95% C.I)	Coefficient (%)	Pct. Contribution
Urban Residence	0.355 (0.34–0.37)*	35.5	–
Rural Residence	0.211 (0.2–0.22)*	21.1	–
Difference (D)	0.144 (0.13–0.16)*	14.4	–
Endowment (E)	0.123 (0.1–0.13)*	12.3	85.4
Coefficient (C)	0.018 (0.01–0.05)*	1.8	12.5
Interaction	0.003 (−0.01–0.02)	0.3	2.1

*-significant at 5%; *C.I* Confidence Interval; *Pct* Percentage

**Table 5:** Detailed decomposition analysis of overweight and obesity among women of reproductive age in Nigeria

Overweight and Obesity	Difference due to characteristics (E)		Difference due to Coefficient (C)	
	Coefficient (95% C.I)	Pct. (%)	Coefficient (95% C.I)	Pct. (%)
**Wealth Status**				
Poor	0.035 (0.028, 0.041)*	24.3	0.0001 (− 0.013, 0.014)	0.01
Middle	−0.0003 (− 0.001, 0.000)	−0.2	− 0.004 (− 0.009, 0.001)	−2.8
Rich	0.035 (0.019, 0.029)*	24.3	0.004 (−0.001, 0.009)	2.7
**Age**				
15–24	0.002 (0.000, 0.005)*	1.4	−0.001 (− 0.011, 0.009)	− 0.7
25–34	0.000 (− 0.000, 0.000)		0.000 (− 0.006, 0.007)	
35–49	0.000 (− 0.002, 0.002)		0.000 (− 0.007, 0.008)	
**Educational Level**				
None	0.023 (0.02, 0.03)*	15.9	0.007 (−0.006, 0.019)	4.9
Primary	0.0003 (−0.000, 0.001)	0.7	0.000 (− 0.004, 0.005)	
Secondary	0.004 (0.001, 0.007)	2.8	−0.003 (− 0.01, 0.004)	−2.1
Tertiary	0.01 (0.007, 0.014)*	6.9	−0.001 (− 0.002, 0.001)	−0.7
**Marital Status**				
Single	− 0.004 (− 0.01, − 0.002)*	−2.8	− 0.000 (− 0.007, 0.007)	
Currently Married	−0.001 (− 0.003, − 0.001)	−0.7	0.004 (− 0.014, 0.021)	2.8
Previously Married	0.001 (0.000, 0.001)*	0.7	−0.000 (− 0.002, 0.002)	
**Media Exposure**				
Not Exposed	0.007 (0.005, 0.011)*	4.9	0.004 (−0.003, 0.011)	2.8
Exposed	0.007 (0.005, 0.011)*	4.9	−0.005 (− 0.014, 0.004)	−3.5
**Contraceptives use**				
None-User	0.003 (0.001, 0.004)*	2.1	−0.004 (− 0.017, 0.010)	−2.8
User	0.003 (0.001, 0.004)*	2.1	0.001 (− 0.001, 0.002)	0.7
Constant	–	–	0.015 (−0.009, 0.039)	10.4

*-significant at 5% (*p*-value < 0.05); *C.I* Confidence Interval; *Pct* Percentage

The endowment effect represents the contribution of women’s characteristics to the disparity. In this analysis, the endowment effect accounted for 10.8% of the overall disparity. This suggests that differences in women’s characteristics explained much of the disparity, specifically 85.4% (0.123/0.144). Factors such as age, educational level, marital status, wealth status, media exposure, and contraceptive use were examined in Table 5 to determine their individual contributions to the endowment effect.

The coefficient effect represents the contribution of the covariates entered in the model to the disparity. It accounted for 1.8% of the overall disparity, indicating an unexplained portion. The coefficient effect examines the specific impact of each variable included in the analysis. Table 5 provides the coefficients and percentages for each variable, demonstrating their contributions to the coefficient effect. Of these characteristics, wealth status and educational level were found to be the most significant contributors to the disparity, with rich, middle, and poor wealth status of women accounting for 24.3% (0.035/0.144), −0.2% (−0.0003/0.144) and 24.3% (0.035/0.144), respectively. The lack of formal education also played a role in contributing to the disparity, with 15.9% of the total attributed to this variable. Addressing the difference in wealth status between rural and urban women would therefore lead to a reduction of approximately 49% in the overall disparity.

Women aged 15–24 women significantly contributed less than 2% of the disparity while exposure to media made a significant contribution having both exposed and unexposed to media accounting for 9.8% of the disparity. Age, use, and non-use of contraceptives contribute 2.1% each to the differences in overweight and obesity between rural and urban residential. The remaining 12.5% of the disparity (0.018/0.144) is due to the differential effect of the covariate entered in the model (i.e., coefficient effect) and the general effect of unknown factors. The coefficient effect suggests an unexplained portion of the disparity, with the lack of formal education having the highest contribution to this component (0.007/0.144 = 4.9%). The rich in wealth status (0.004/0.144 = 2.7%), while not statistically significant, also contributed to this component. Conversely, poor and middle wealth statuses, as well as secondary and tertiary education level of education were found to have a negative contribution to this component, which would widen the disparity upon removal of rural-urban differences in these variables. Also, being single and currently married was found to have a negative contribution to the differences due to the women’s endowment component.

The interaction effect captures the combined influence of the endowment and coefficient effects. In this analysis, the interaction effect was not statistically significant, suggesting that the interaction between these effects did not contribute significantly to the overall disparity.

## Discussion

While researchers have widely recognized the increasing prevalence of overweight and obesity as a significant public health challenge, effective interventions in developing countries are hindered by a lack of high-quality information required to understand the exact situation. The literature review has revealed a scarcity of evidence regarding how the sociodemographic characteristics of women in Nigeria contribute to overweight and obesity among women of reproductive age. To address this gap, this study utilized DHS data to dissect the factors associated with overweight and obesity among women of reproductive age in Nigeria. Initially, we examined the association between sociodemographic factors and overweight and obesity while controlling for other variables. Furthermore, we employed the Blinder Oaxaca decomposition model to identify and quantify the contributions of group differences in factors affecting overweight and obesity.

## Determinants of overweight and obesity by sociodemographic characteristics of women

The logistic regression analysis results showed that household poverty-wealth contributes to being overweight and obese. Specifically, our finding shows that women who belong to wealthy households are more at risk of being overweight or obese. Similarly, our study shows in the unadjusted logistic regression analysis that the risk of being overweight and obese is twice as high among women who are employed compared to unemployed women. These findings corroborate findings from previous studies on overweight and obesity in Nigeria [18, 36]. In most developing nations, household wealth is a predictor of overweight and obesity. The relationship between wealthy households and overweight/obesity can be attributed to having sedentary lifestyles and various diets that are high in energy [36]. On the other hand, women belonging to poor households are more prone to malnutrition, caused by poor food intake such as protein-energy malnutrition, vitamin deficiencies, and diet-related, which may lead to being underweight [18]. Another plausible explanation is that women from wealthy households are more likely to have high incomes to afford diets rich in saturated fats. Moreover, overweight/obese are culturally associated with wealth and sexual attractiveness in Nigeria, particularly in women [36].

Consistent with a previous study in Nigeria, our results indicated that the prevalence and risk of overweight and obesity are higher among older women compared to young women [36]. Age-related hormonal changes, a decline in physical activities, and an increase in energy-dense food consumption, as people get older, are probably contributing to the prevalence and risk of overweight and obesity. On the other hand, younger women are expected to be more interested in their health and participate in more physical activities than older women [36].

Findings from this study revealed that the prevalence and the risk of overweight and obesity are highest among women with higher education. These findings are consistent with previous studies [12]. The observed association between overweight and obesity and higher education may arise from either a sedentary lifestyle or the occupational characteristics typically associated with individuals possessing higher levels of education. These characteristics are often characterized by lower levels of physical activity when compared to individuals with lower educational attainment. To further explain this association, it could be because people with more education often have jobs that involve sitting at a desk or using a computer for most of the day. This kind of work doesn’t require much physical movement. It might also be because people with less education often have jobs that involve more physical activity, like construction or farming. So, the level of education can affect the kind of work people do, and that, in turn, can influence how active they are. Nevertheless, the mechanism by which education is associated with overweight and obesity among women has spawned a variety of discussions in Nigeria. For instance, a previous study [45] conducted in Nigeria reported that women with at least primary education have greater odds of being overweight and obese than any other education category.

Consistent with the findings of Tagbo, Abebe, and Oguoma (2021) [35], our study revealed that the odds of being overweight/obese are higher among women who are currently or previously married. A plausible explanation could be linked to the fact that married women may engage in less physical activity, have consistent dietary patterns, and may be affected by metabolic changes from postpartum [11]. Our finding also revealed that the prevalence and the risk of overweight/obesity increases as the number of living children by women increases. The finding on the association between the number of children ever born and overweight/obesity corroborates the finding of a study conducted in Zambia, which revealed that the odds of being overweight/obese are higher among women who have 4–6 two children [7]. The logistic regression analysis demonstrates that contraceptive use is an important determinant of overweight/obesity among women. This finding confirms the finding of a previous study in Ethiopia, which reported a positive association between contraceptive use and overweight/obesity [46]. A plausible explanation for the relationship between contraceptive use and overweight/obesity may be linked to the hormonal effect of contraceptives that contributes to weight gain [47].

We discovered in our logistic regression analysis that women who live in rural areas have a lower risk of being overweight/obese, which is in line with previous studies in Nigeria [18, 36, 45]. These inequalities between the urban and rural areas in Nigeria may be explained by variances in urban people’s lifestyles, nutritional habits, technological advancements, and more prevalence of vehicles that limit mobility exercises. Fast food consumption has become normalized in urban areas and is seen as a show of wealth, leading to a larger intake of items that are higher in energy than in rural areas—in addition to increased food intake, urbanization, and technological development led to a transition from manual labor to more sedentary jobs and a decline in physical activity. However, residents of rural areas typically practice non-mechanized farming and consume nutritious, locally produced foods. Our findings also revealed that North Central, North East, and North West all have lower prevalence and risks of overweight/obesity compared to the rest regions of Nigeria. A possible explanation is that the Northern regions of Nigeria have lower socioeconomic status (SES) than the Southern regions, which have rapid urbanization and higher SES, which may lead to a higher risk of overweight/obesity. Moreover, women in the Northern regions are more likely to engage in greater physical activity since they predominantly work in agricultural, farming, and pastoral sectors.

Contrary to our findings, a previous study on geographical variation of overweight and obesity among women in Nigeria documented no association between religion and overweight/obesity. Specifically, our study found that Muslims and other religions have a lower risk of overweight/obesity compared to Christians. Religion has its own set of lifestyle guidelines and practices which may have effects on eating, drinking, and smoking habits. Moreover, some religious practices such as the Muslims involve a change of body positions during prayers, which some regard as exercise, which may lower the risk of being overweight/obese. Consistent with a previous study in Nigeria, our study revealed that being exposed to media increases the risk of overweight/obesity among women in Nigeria [34]. The main underlying mechanism for this association is that prolonged and frequent usage of sedentary leisure activities such as social media leads to increased exposure to food advertisements, overeating, extended times of rest, decreased energy expenditure, and subsequent weight gain [34].

## The rural-urban disparity in overweight and obesity is explained by the sociodemographic characteristics of women

The influence of residential location on health outcomes is profoundly significant. Urban areas, characterized by a high concentration of fast-food establishments, convenience stores, and modern technologies facilitating the availability of processed foods, contribute to an environment conducive to poor health [18, 36]. The sedentary lifestyle prevalent in urban settings, resulting from limited opportunities for physical activity due to crowded living conditions and a scarcity of open spaces, further exacerbates the issue [12]. These factors may help clarify the higher prevalence of overweight and obesity among urban women, as evidenced by the findings of this study. Urban environments offer residents easier access to calorie-dense and unhealthy food options, which, coupled with higher levels of chronic stress, can lead to emotional eating and unhealthy coping mechanisms [45]. Stressors in urban settings, such as noise pollution, overcrowding, and social isolation, can detrimentally affect mental well-being and contribute to an elevated risk of overweight and obesity [7, 11, 47].

In contrast, rural areas may have limited availability of affordable and diverse food choices. However, they often rely on traditional, home-cooked meals prepared using fresh, locally sourced ingredients, which are generally considered healthier. The findings of this study align with previous research conducted in various regions of Africa and worldwide, reinforcing the notion that environmental factors influence the prevalence of overweight and obesity [11–47].

This study explores the disparities in overweight and obesity between rural and urban populations, with a focus on the influence of women’s characteristics. Specifically, factors such as wealth status, educational level, media exposure, and contraceptive use were identified as significant contributors to these disparities. This finding aligns with previous studies conducted in the field, indicating the consistent influence of socioeconomic factors on the observed differences between rural and urban communities [37, 47].

Urban areas often have a higher concentration of low-income communities and marginalized populations, which can limit their access to nutritious food, healthcare, and educational resources. The prevalence of advertisements promoting unhealthy food choices on social media platforms in urban areas, in contrast to the stronger cultural traditions and practices promoting healthier eating patterns and active lifestyles in rural settings, may further explain the high rural-urban disparity [33]. It is important to note that while urban areas generally offer better access to healthcare facilities and services, disparities can still exist within these settings. Factors such as inadequate healthcare infrastructure, limited availability of specialized care, and insufficient access to preventive services can hinder effective obesity management and contribute to poorer health outcomes among urban populations.

The prevalence of overweight and obesity in urban areas can be indirectly influenced by educational attainment through various mechanisms. A higher level of education is generally associated with greater health knowledge and awareness. Individuals with more education often have a better understanding of the importance of maintaining a healthy diet, engaging in regular physical activity, and the potential risks associated with overweight and obesity. Consequently, they are more likely to adopt health-promoting behaviors, leading to lower rates of overweight and obesity. However, it is important to acknowledge that the rural-urban disparity observed in this study may arise from the existence of highly marginalized and less privileged segments of the urban population who lack formal education. These individuals may face significant barriers to accessing health information and resources, which can contribute to higher rates of overweight and obesity among them. These findings are consistent with earlier studies, emphasizing the critical role of education and wealth in addressing the issue of overweight and obesity [21, 32, 39, 47].

## Study limitations and strengths

This study contributes to the knowledge of overweight and obesity disparities by the rural-urban status of women in Nigeria. However, there are a few limitations to be considered when interpreting the findings of this study. First, the cross-sectional study design of the DHS made it impossible to assert causation. Second, there might be possibilities of bias because of the many factors that were self-reported. Third, insufficient data on weight and height for women limited our unit of analysis. Nonetheless these limitations, this study offers valuable insights into the ongoing discussion regarding disparities in overweight and obesity among women. Furthermore, the study utilized a nationally representative sample which allows inference to the entire population of Nigeria.

## Conclusion

This study illuminates the multifaceted factors that predispose women to being overweight and obese. It also sheds light on the various characteristics of women to the observed differences in overweight and obesity between rural and urban areas. In conclusion, the rural-urban disparity in overweight and obesity among women in Nigeria highlights the need for focused public health interventions and policies when examined through the prism of some important variables. This inequality is not solely attributed to geographical location; it is also significantly influenced by a few other variables, such as wealth status and education, age, media exposure, and contraceptive use. The findings of this study underscore the significance of tackling the underlying causes of overweight and obesity in both urban and rural areas and not just the conditions themselves.

Interventions that are specifically designed to meet the needs and overcome the obstacles of overweight and obese Nigerian women in rural and urban areas are essential. Since every setting has its own set of possibilities and constraints, efforts to close this gap should be diverse. Increasing women’s empowerment, advancing nutrition education, and encouraging a culture of physical exercise should all be part of the strategy.

## Data Availability

The study used secondary data from dhsprogram.org, which is publicly available at https://dhsprogram.com/data/available-datasets.cfm.

## References

[1] WHO. Obesity. Fact sheets. 2021. [cited 2023 June 6]. Available from: https://www.who.int/news-room/fact-sheets/detail/obesity-and-overweight.

[2] Kelly T, Yang W, Chen CS, Reynolds K, He J (2008). Global burden of obesity in 2005 and projections to 2030. Int J Obes..

[3] Agyemang C, Boatemaa S, Frempong GA, Aikins A de-G. Obesity in Sub-Saharan Africa 2016;41–53.

[4] Okoh M (2013). Socio-demographic correlates of overweight and obesity among women of reproductive age in Nigeria on JSTOR. African J Reprod Heal..

[5] Adaja T, Idemudia O (2018). Prevalence of overweight and obesity among health-care workers in University of Benin Teaching Hospital, Benin City, Nigeria. Ann Trop Pathol..

[6] CDC (2021). About overweight and obesity | obesity | DNPAO |.

[7] Bwalya BB, Mapoma CC, Mulenga JN. Rural-urban differentials in the prevalence of overweight and obesity among women of child bearing age in Zambian. Epidemiol Biostat Public Heal. 2017;14(2)

[8] Ajayi K, Alli YR, Taiwo OM (2015). Prevalence of obesity among urban and rural dwellers in Nigeria. J Nutr Health Food Eng..

[9] Beslay M, Srour B, Méjean C, Allès B, Fiolet T, Debras C, Touvier M (2020). Ultra-processed food intake in association with BMI change and risk of overweight and obesity: a prospective analysis of the French NutriNet-Santé cohort. PLoS Med..

[10] Chooi YC, Ding C, Magkos F (2019). The epidemiology of obesity. Metabolism..

[11] Larson NI, Story MT, Nelson MC. Neighborhood environments: disparities in access to healthy foods in the U.S. Am J Prev Med. 2009;36(1) 10.1016/j.amepre.2008.09.025 18977112

[12] Krefis A, Augustin M, Schlünzen K, Oßenbrügge J, Augustin J (2018). How does the urban environment affect health and well-being? A systematic review. Urban Sci..

[13] Mahumud RA, Sahle BW, Owusu-Addo E, Chen W, Morton RL, Renzaho AM (2021). Association of dietary intake, physical activity, and sedentary behaviours with overweight and obesity among 282,213 adolescents in 89 low and middle income to high-income countries. Int J Obes..

[14] Goodarzi MO (2018). Genetics of obesity: what genetic association studies have taught us about the biology of obesity and its complications. The Lancet Diab Endocrinol..

[15] Chen Y, Liu X, Yan N, Jia W, Fan Y, Yan H, Ma L (2020). Higher academic stress was associated with increased risk of overweight and obesity among college students in China. Int J Environ Res Public Health..

[16] Pereira-Miranda E, Costa PR, Queiroz VA, Pereira-Santos M, Santana ML (2017). Overweight and obesity associated with higher depression prevalence in adults: a systematic review and meta-analysis. J Am Coll Nutr..

[17] Mangemba NT, San SM (2020). Societal risk factors for overweight and obesity in women in Zimbabwe: a cross-sectional study. BMC Public Health..

[18] Van Kamp I, Van Loon J, Droomers M, De Hollander A (2021). Residential environment and health: a review of methodological and conceptual issues. Rev Environ Health..

[19] Ogbuji Q (2010). Obesity and reproductive performance in women - PubMed. Afr J Reprod Health..

[20] Dağ ZÖ, Dilbaz B. Impact of obesity on infertility in women. J Turk German Gynecol Assoc AVES Ibrahim Kara. 2015;16:111–7. 10.5152/jtgga.2015.15232 PMC4456969 26097395

[21] Al-Qahtani AM. Prevalence and predictors of obesity and overweight among adults visiting primary care settings in the southwestern region, Saudi Arabia. Biomed Res Int. 2019;2019 10.1155/2019/8073057 PMC6425323 30949511

[22] Ziraba AK, Fotso JC, Ochako R (2009). Overweight and obesity in urban Africa: a problem of the rich or the poor?. BMC Public Health..

[23] Raymond SU, Leeder S (2006). Greenberg HM. Obesity and cardiovascular disease in developing countries: A growing problem and an economic threat. Curr Opin Clin Nutr Metab Care..

[24] WHO. WHO | global status report on noncommunicable diseases 2014. WHO. 2015; 10.1161/STROKEAHA.115.008097 25873596

[25] Kulie T, Slattengren A, Redmer J, Counts H, Eglash A, Schrager S (2011). Obesity and women's health: an evidence-based review. The J Am Board Fam Med..

[26] Dabelea D, Harrod CS. Role of developmental overnutrition in pediatric obesity and type 2 diabetes. Nutr Rev. 2013;71(SUPPL1) 10.1111/nure.12061 24147926

[27] Parnell AS, Correa A, Reece EA (2017). Pre-pregnancy obesity as a modifier of gestational diabetes and birth defects associations: a systematic review. Matern Child Health J..

[28] Finkelstein EA, Graham WCK, Malhotra R (2014). Lifetime direct medical costs of childhood obesity. Pediatrics..

[29] Abdelaal M, le Roux CW, Docherty NG (2017). Morbidity and mortality associated with obesity. Annals of Translational Medicine.

[30] Wang YC, McPherson K, Marsh T, Gortmaker SL, Brown M (2011). Health and economic burden of the projected obesity trends in the USA and the UK.

[31] Nishida C, Borghi E, Branca F, et al. Global trends in overweight and obesity. In: Romieu I, Dossus L, Willett WC, editors. Energy Balance and Obesity. Lyon (FR): International Agency for Research on Cancer; 2017. (IARC Working Group Reports, No. 10). Available from: https://www.ncbi.nlm.nih.gov/books/NBK565817/. 33534469

[32] Kandala NB, Stranges S. Geographic variation of overweight and obesity among women in Nigeria: a case for nutritional transition in sub-Saharan Africa. PLoS One. 2014;9(6) 10.1371/journal.pone.0101103 PMC4076212 24979753

[33] Alaba O, Chola L (2014). Socioeconomic inequalities in adult obesity prevalence in South Africa: a decomposition analysis. Int J Environ Res Public Health..

[34] Hasan E, Khanam M, Shimul SN. Socio-economic inequalities in overweight and obesity among women of reproductive age in Bangladesh: a decomposition approach. BMC Womens Health. 2020;20(1) 10.1186/s12905-020-01135-x PMC7691075 33243211

[35] Tagbo SO, Abebe D, Oguoma VM (2021). Overweight and obesity among non-pregnant women of reproductive age in Nigeria: findings from the 2008–2018 Nigerian demographic and health survey. Public Health..

[36] Westbury S, Ghosh I, Jones HM, Mensah D, Samuel F, Irache A (2021). The influence of the urban food environment on diet, nutrition and health outcomes in low-income and middle-income countries: a systematic review the influence of the urban food environment on diet, nutrition and health outcomes in low-income and middle-income countries: a systematic review. BMJ Global BMJ Glob Heal..

[37] Kumar P, Mangla S, Kundu S (2022). Inequalities in overweight and obesity among reproductive age group women in India: evidence from National Family Health Survey (2015–16). BMC Womens Health..

[38] Mengesha Kassie A, Beletew Abate B, Wudu KM (2020). Education and prevalence of overweight and obesity among reproductive age group women in Ethiopia: analysis of the 2016 Ethiopian demographic and health survey data. BMC Public Health..

[39] Stoltzfus JC (2011). Logistic regression: a brief primer. Acad Emerg Med..

[40] Boateng EY, Abaye DA, Boateng EY, Abaye DA (2019). A review of the logistic regression model with emphasis on medical research. J Data Anal Inf Process..

[41] Fairlie RW (2005). An extension of the Blinder-Oaxaca decomposition technique to logit and probit models 1. J Econ Soc Measure..

[42] Rahimi E, Hashemi Nazari SS (2021). A detailed explanation and graphical representation of the blinder-Oaxaca decomposition method with its application in health inequalities. Emerg Themes Epidemiol..

[43] Jann B (2008). The blinder-Oaxaca decomposition for linear regression models. Stata J..

[44] Jann B. Decomposition methods in the social sciences. 2018. [cited 2023 April 16]. Available from https://boris.unibe.ch/117107/49/1-intro.pdf.

[45] Bivoltsis A, Trapp G, Knuiman M, Hooper P, Ambrosini GL (2020). The influence of the local food environment on diet following residential relocation: longitudinal results from RESIDential environments (RESIDE). Public Health Nutr..

[46] Endalifer ML, Diress G, Addisu A, Linger B (2020). The association between combined oral contraceptive use and overweight/obesity: a secondary data analysis of the 2016 Ethiopia demographic and health survey. BMJ Open..

[47] Hashan MR, Das Gupta R, Day B, Al Kibria GM. Differences in prevalence and associated factors of underweight and overweight/obesity according to rural–urban residence strata among women of reproductive age in Bangladesh: evidence from a cross-sectional national survey. BMJ Open. 2020;10(2) 10.1136/bmjopen-2019-034321 PMC7045126 32024791

